# Rapid maturation of the hepatic cell line Huh7 via CDK inhibition for PXR dependent CYP450 metabolism and induction

**DOI:** 10.1038/s41598-019-52174-w

**Published:** 2019-11-01

**Authors:** Beyza Bulutoglu, Safak Mert, Camilo Rey-Bedón, Sarah L. Deng, Martin L. Yarmush, O. Berk Usta

**Affiliations:** 1 Center for Engineering in Medicine, Massachusetts General Hospital, Harvard Medical School and Shriners Hospitals for Children, Boston, MA 02114 USA; 2 Department of Biomedical Engineering, Rutgers University, Piscataway, NJ 08854 USA

**Keywords:** Biotechnology, Toxicology, Cell biology

## Abstract

CYP3A4, a cytochrome P450 enzyme regulated by the nuclear receptor PXR, is involved in most of the drug metabolizing pathways. Studying the regulation/induction of CYP3A4 and PXR is critical in toxicology and drug-drug interaction (DDI) studies. Primary human hepatocytes constitute the preferred *in vitro* platform for drug development efforts. However, they are expensive, scarce and heterogeneous. Hepatic cell lines, such as Huh7, could provide a cost-effective alternative, however, they express negligible amounts of CYP450s and PXR. In this study, we show that dinaciclib, a potent cyclin dependent kinase inhibitor, significantly increases the basal CYP3A4 and PXR levels in 24 hours. We also demonstrated that matured Huh7s can be used for drug induction studies, where CYP3A4, CYP1A2, CYP2C9, and CYP2C19 inductions were achieved following rifampicin treatment. More importantly, through a direct demonstration using amiodarone and rifampicin as model drugs, we showed that matured Huh7s present a suitable platform for DDI studies.

## Introduction

Liver is the primary organ responsible for the metabolism and detoxification of the vast majority of xenobiotics, including pharmaceuticals^1,2^. Hepatocytes, the parenchymal cells of the liver, express nuclear receptors, like pregnane X receptor (PXR; NR1I2), and constitutive androstane receptor (CAR; NR1I3), which are allosterically activated upon interactions with xenobiotics^3,4^. When activated, these receptors regulate the expression of many downstream genes responsible for the detoxification of xenobiotics^3,5,6^. The expression of many phase I, II, and III enzymes, and transporters, e.g. efflux pumps like multidrug resistance proteins, responsible for detoxification and removal of xenobiotics are controlled by these nuclear receptors^6,7^.

Cytochrome P450 (CYP450) oxidases constitute the main enzymatic system responsible for the phase I metabolism of xenobiotics in hepatocytes^8,9^. CYP3A4, a significantly expressed enzyme among these CYP450s, is responsible for the metabolism of over 50% of all the drugs in clinical use^10^. Drugs that inhibit CYP3A4 activity may lead to increased levels of other co-administered drugs normally detoxified by CYP3A4. This kind of drug-drug interactions (DDI) may lead to the toxic accumulation of certain drugs. On the contrary, any drug capable of increasing CYP3A4 expression may lead to faster metabolization of other drugs, and decrease their efficacy^8,11^, and in some instances result in increased toxicity to the liver and other tissues through intermediate metabolites^12,13^. Thus, the inhibition/induction of CYP3A4 by one drug can severely alter both the efficacy and safety of another co-administered drug. Because of this centrally important role of CYP3A4 in DDI and toxicology studies, it is important to assess both the basal CYP3A4 metabolism and the CYP3A4 inhibiton or induction potential of new drugs under development. Similarly, PXR expression - the key regulator of CYP3A4 expression - is also vital for DDI and toxicology studies^14–17^.

To this end, *in vitro* cultures of primary human hepatocytes (PHHs) are the preferred platform for drug development, toxicology, and DDI studies. Nevertheless, PHHs are usually isolated from marginally healthy donor livers rejected for transplantation. Moreover, PHHs present a significant genetic and phenotypic variability, which may further complicate the outcome of *in vitro* drug studies. While animal derived cells, e.g. rodent hepatocytes, provide a more robust, economical and less variable source; they rarely provide similar outcomes with human-origin cells, due to the differences in their highly complex nature of the xenobiotic response system^18,19^. Animal models are an alternative platform for drug studies, however they can be inadequate in terms of predicting the human response to xenobiotics^20–22^. Another alternative is human-origin cell lines, which are either derived from cancer cells or immortalized from primary cells through various methods. As a result, their gene expression profile, metabolism, and even chromosome organization are substantially different than the primary cells of the originated tissue. Similarly, many hepatic cell lines express negligible amounts of PXR and/or CYP450 enzymes. Despite these shortcomings, cell-lines are easy to use, cheap, readily available, and can be passaged virtually indefinitely. Hence, the development of a cell line with functional xenobiotic response resembling that of PHH would be of interest.

Huh7 is a hepatocellular carcinoma derived cell line of human origin^23^. Huh7 exhibits some characteristics of PHHs and has found usage in hepatocyte-specific virus studies^24–26^. It has been shown that the low to negligible PXR and CYP3A4 expression in Huh7s can be remarkably increased by culturing them over-confluently for 4 weeks^27^. While this is a substantial improvement for the use of Huh7s in drug metabolism studies, this process is time-consuming and thus not well suited to many work-flows. Any approach that can significantly decrease this 4-week maturation period could lead to a practically useful cell line for DDI and drug-induced toxicology studies. CDK2, a member of the cyclin dependent kinase (CDK) family known for their role in regulating the cell cycle, has previously been shown to control the activity of PXR^28^. This study found that the reduction in CDK2 expression correlated well with increases observed in both PXR and CYP3A4 expression during the 4-week confluent maturation of the Huh7 cells. A more recent study applied siRNA mediated CDK2 silencing on Huh7s^29^, albeit with limited success in CYP3A4 induction.

In this study, we tested 4 potent CDK2 inhibitors to induce PHH drug metabolism characteristics in Huh7 cell line in a relatively short 24-hour period. We found that, out of these 4 inhibitors, the effect of dinaciclib on the basal CYP3A4 and PXR expression was better than that of 4-week confluent cultures. In addition, through the significant CYP induction with rifampicin in the dinaciclib pre-treated cells, we demonstrated that this approach allowed for PXR-dependent induction studies in matured Huh7s. Furthermore, we showed the applicability of this approach by demonstrating a CYP3A4 mediated DDI via treatment of matured Huh7s with rifampicin and amiodarone. Taken together, this rapid maturation of CYP3A4/PXR metabolism in Huh7s via dinaciclib treatment can be utilized to generate a feasible and cost-effective platform to conduct various drug metabolism, DDI, and toxicity studies.

## Results

### Basal CYP3A4 and PXR levels increase upon dinaciclib treatment

**Figure 1:**
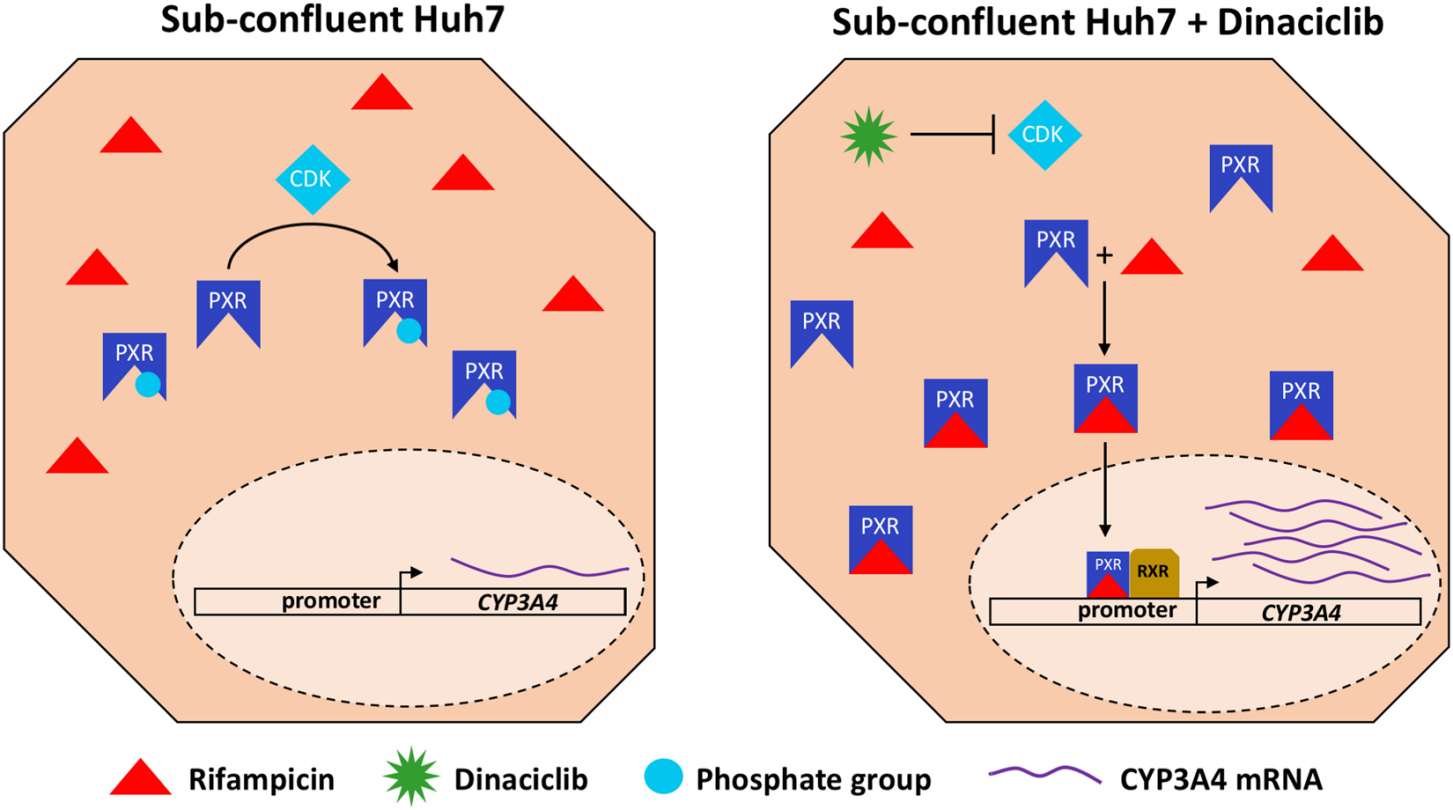
Cartoon illustration of the restoration of the rifampicin induction mechanism by dinaciclib in Huh7 cells. Actively proliferating Huh7 cells express cyclin dependent kinases (CDKs) and CDK2 was shown to phosphorylate PXR, impairing its ligand binding ability. Upon dinaciclib treatment, CDKs are inhibited allowing for PXR to be activated by rifampicin. Following translocation into the nucleus, activated PXR/RXR complex binds to the promoter region of CYP3A4, inducing its transcription.

Previously, CDK2 pathway inhibition via cell-to-cell contact in 4-week confluent Huh7 cultures was shown to result in an increase in basal PXR and CYP3A4 expression levels^27^. In this study, we have tested 4 different known CDK inhibitors: aloisine A, BML-259, CDK1/2 inhibitor III and dinaciclib in order to achieve an increase in the concentration of inducible PXR molecules, as demonstrated in Fig. 1. Based on their established IC50 values, we incubated Huh7s with two different concentrations of each inhibitor for 24 hrs to achieve both observable and saturated inhibition levels. The changes in basal CYP3A4 and PXR expression levels were quantified via qPCR following the treatment as demonstrated in Figure S1. We observed statistically significant increases in basal expression levels for both proteins only for the dinaciclib treated group. CYP3A4 expression was upregulated by 11 and 24-fold whereas PXR levels increased by 8 and 19-fold, following the 24 hrs treatment with 10 nM and 20 nM dinaciclib, respectively.

### Dose-response and change in basal expression levels

**Figure 2:**
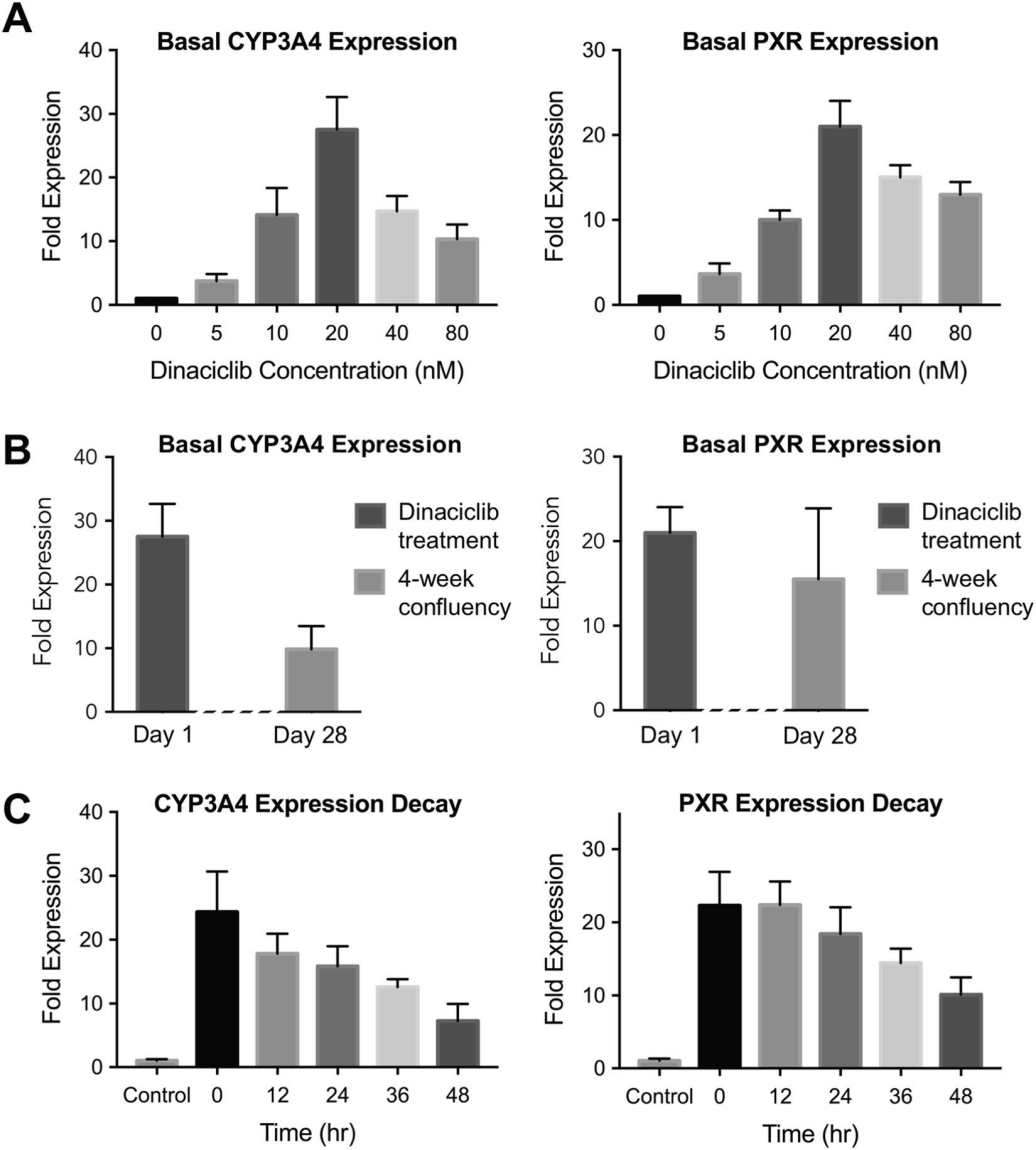
Effectiveness of dinaciclib treatment on CYP3A4 and PXR expression levels. Dinaciclib dose response and time response studies. (**A**) A dose response study was conducted where Huh7 cells were treated with different dinaciclib concentrations for 24 hrs. The fold changes in expression levels of CYP3A4 and PXR are shown. (**B**) Comparison of the effect of 4-week confluency versus dinaciclib treatment on the basal expression levels of CYP3A4 and PXR. In 4-week confluent cultures, CYP3A4 and PXR levels were quantified via quantitative real-time PCR and plotted as fold expression normalized to freshly plated Huh7s. The other group was freshly plated and treated with 20 nM dinaciclib for 24 hrs. CYP3A4 and PXR expression levels were quantified in the same way. (**C**) A time response study was conducted where Huh7s were treated with 20 nM dinaciclib for 24 hrs. Following the treatment, the fold-changes in CYP3A4 and PXR mRNA levels were quantified via quantitative real-time PCR at different time points, up to 48 hrs. The data are presented as mean ± SEM, N = 6.

After identifying dinaciclib as a promising candidate to increase the basal CYP3A4 and PXR expression levels, we performed a more detailed dose-response study to assess the efficacy of this CDK inhibitor with a dose range of 0–80 nM. Following 24-hrs incubation, the fold change in basal expression levels showed that maximum expression (28 and 21-fold for CYP3A4 and PXR, respectively) was achieved at 20 nM dinaciclib treatment. Further increases in dinaciclib concentration, i.e. 40 and 80 nM, resulted in higher than basal CYP3A4 and PXR levels but lower than those with 20 nM dinaciclib treatment (Fig. 2A).

As a comparison, we cultured Huh7s confluently for 4 weeks and quantified the fold change in basal CYP3A4 and PXR expression levels compared to freshly plated cultures. We compared these results to the 20 nM dinaciclib treated group, as shown in Fig. 2B. Basal CYP3A4 and PXR levels were upregulated by 9.8-fold and 15.5-fold in 4-week confluently cultured cells. This data demonstrated that dinaciclib treatment for 24 hrs resulted in higher basal CYP3A4 and PXR levels, compared to 4-week cultures and that rapid maturation of CYP3A4/PXR metabolism in Huh7s can be achieved via dinaciclib treatment, decreasing the maturation timeline from 4 weeks to 1 day.

As the next step, we performed a time-course study where we investigated CYP3A4 and PXR expression levels at different time points (up to 48 hrs) following treatment with 20 nM dinaciclib for 24 hrs (Fig. 2C). We observed a gradual decay in basal expression levels of both proteins where CYP3A4 and PXR induction levels decreased to 16-fold and 18-fold, respectively, at the end of 24 hrs after dinaciclib treatment. At the end of 48 hrs, the dinaciclib treatment was still effective with a 7 and 10-fold expression level, for CYP3A4 and PXR, respectively, compared to the non-treated group.

### Dinaciclib pre-treatment allows for rifampicin induction in Huh7s

**Figure 3:**
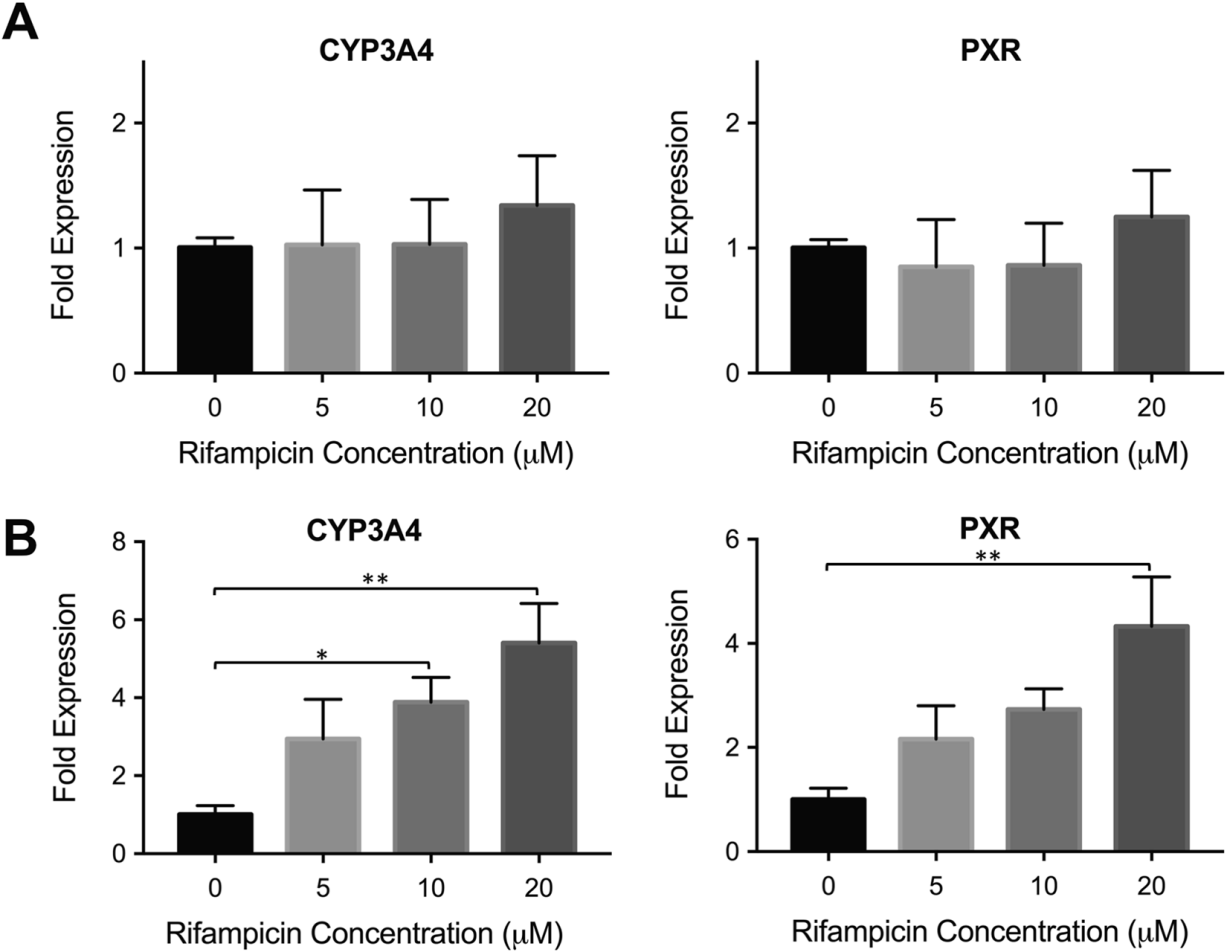
Rifampicin induction in dinaciclib pre-treated Huh7s. (**A**) Non-treated Huh7 cells were incubated with 0 μM, 5 μM, 10 μM and 20 μM rifampicin, an inducer of CYP3A4 through PXR, for 24 hrs. The mRNA levels of CYP3A4 and PXR were quantified via quantitative real-time PCR. (**B**) Dinaciclib pre-treated Huh7 cells were incubated with the same rifampicin concentrations. A gradual increase in fold-expression levels of CYP3A4 and PXR is observed. The data are presented as mean ± SEM. N = 6, *p ≤ 0.05, **p ≤ 0.01.

After confirming that dinaciclib treatment increases basal PXR expression and subsequent CYP3A4 expression, we performed enzymatic induction studies to assess the suitability of dinaciclib pre-treated Huh7s for use in drug-enzyme and drug-drug interaction studies. To this end, Huh7s, non-treated and pre-treated with 20 nM dinaciclib for 24 hrs, were incubated with varying concentrations of rifampicin (5–20 μM), a well-known activator of PXR and inducer of CYP3A4^30^. At the end of 24 hr incubation time, cells were lysed, and CYP3A4 and PXR expression levels were quantified as shown in Fig. 3.

No statistically significant changes in mRNA levels were observed for the non-treated group, showing that Huh7s did not respond to rifampicin treatment (Fig. 3A). On the other hand, a dose-dependent increase in CYP3A4 fold expression levels was achieved in the dinaciclib pre-treated group upon induction with increasing rifampicin concentrations. At 10 μM and 20 μM rifampicin, the CYP3A4 levels were significantly induced by 3.9-fold and 5.4-fold, respectively, as shown in Fig. 3B. A gradual increase in PXR mRNA levels was determined as well, although statistically significant expression levels were observed at 20 μM rifampicin only, where PXR levels were upregulated by 4.3-fold, compared to the non-treated control group.

As a comparison, we performed induction studies with 4-week confluently cultured Huh7s. These cells were treated with varying concentrations of rifampicin after the 4 weeks confluent culturing period. The mRNA levels showed that CYP3A4 and PXR levels were induced by 4.9-fold and 3.6-fold, respectively, whereby statistically significant upregulation was achieved for CYP3A4 only (Fig. S2). These results, together with the data presented in Fig. 2B, demonstrate that the dinaciclib pre-treatment allowed for the maturation of this hepatic cell line within 24 hrs. This rapid maturation approach results in higher basal expression levels for CYP3A4 and PXR compared to 4-week confluent cultures, and allows for rifampicin inducibility, where higher induction levels were obtained in the case of the dinaciclib pre-treated group.

### CYP enzyme inductions in Huh7s pre-treated with dinaciclib

We showed that dinaciclib treatment allowed for CYP3A4 induction via rifampicin. We then investigated if the induction of other PXR-dependent CYPs can be studied via this improved cell line-based platform. We studied the expression levels of CYP1A2, 2C9, and 2C19 upon rifampicin treatment in non-treated and pre dinaciclib-treated Huh7s. The expression levels of these three CYP enzymes are also regulated by PXR. In addition to these, we also quantified the expression levels of CYP2E1, which is not regulated by PXR.

**Figure 4:**
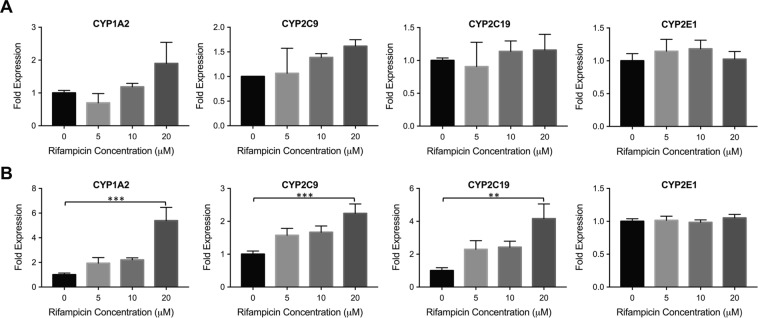
CYP inducibility in dinaciclib pre-treated Huh7s. (**A**) Non-treated Huh7 cells were incubated with increasing rifampicin concentrations for 24 hrs. The mRNA levels of CYP1A2, CYP2C9, CYP2C19, and CYP2E1 were quantified via quantitative real-time PCR. Out of these four enzymes, the first three are regulated by PXR whereas CYP2E1 is not. (**B**) The same experiment was performed in dinaciclib pre-treated Huh7 cells. The data are presented as mean ± SEM. N = 6, **p ≤ 0.01, ***p ≤ 0.001.

Figure 4 demonstrates the change in fold expression levels of these four CYP enzymes upon rifampicin treatment in non-treated (Fig. 4A) and dinaciclib pre-treated (Fig. 4B) Huh7s. The mRNA levels of 1A2, 2C9 and 2C19 were upregulated upon rifampicin induction in the dinaciclib pre-treated group. Following 20 μM rifampicin treatment, 5.4-, 2.3- and 4.2-fold increases were observed for the expression levels of 1A2, 2C9 and 2C19, respectively. In the non-treated group, the expression levels of these enzymes did not change significantly upon rifampicin induction (Fig. 4A). CYP2E1 expression levels were not altered, neither in the dinaciclib pre-treated nor the non-treated groups, demonstrating the specificity of dinaciclib treatment for the improvement of PXR-mediated CYP450 enzyme induction.

### Dinaciclib pre-treatment allows for DDI studies in Huh7s

**Figure 5:**
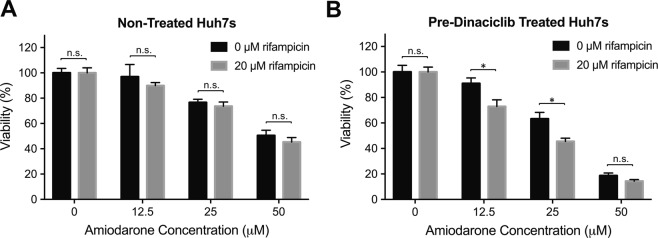
Demonstration of the applicability of dinaciclib pre-treated Huh7s for DDI studies via induction of rifampicin-amiodarone interaction. (**A**) Non-treated Huh7 cells were incubated with increasing amiodarone concentrations in the absence or presence of rifampicin. Following 24 hrs of incubation, the cell viability was assessed via PrestoBlue assay. (**B**) The same experiment was performed in dinaciclib pre-treated Huh7s. The data are presented as mean ± SEM. N = 6, n.s. = not significant, *p ≤ 0.05.

Having established a practical cellular platform for enzymatic induction via the fast maturation of the Huh7s, we demonstrated their applicability directly in DDI studies. To this end, we chose amiodarone, another drug that is also metabolized by CYP3A4, and was shown to result in cell death in the presence of CYP3A4-inducer rifampicin^31^. We incubated the non-treated and dinaciclib treated Huh7s with different concentrations of amiodarone (0 μM, 12.5 μM, 25 μM and 50 μM) in the absence or presence of 20 μM rifampicin to assess the interaction of rifampicin with amiodarone and its effect on cell health. Following incubation for 24 hrs, we assessed the cell viability via PrestoBlue assay, as shown in Fig. 5. In non-treated cells, we did not observe a statistically significant change between the 0 μM and 20 μM rifampicin groups (Fig. 5A). However, in the dinaciclib pre-treated group, cell viability was significantly lower in the rifampicin treated group at amiodarone concentrations of 12.5 μM and 25 μM (Fig. 5B), indicating the DDI between the two drugs following the activation of PXR/CYP3A4 metabolism via dinaciclib treatment. Furthermore, in the absence of rifampicin, the cell viability was also lower in the dinaciclib-treated group compared to the non-treated group, as increased CYP3A4 concentrations resulted in higher concentrations of toxic amiodarone metabolites.

## Discussion

Hepatic cell lines are often used in place of primary human hepatocytes as they are cheap, immortal and easy to access. However, they lack the functional xenobiotic response, particularly CYP450 metabolism, renders them impractical for toxicology and DDI investigations. In this study, we developed a new method to rapidly mature the CYP3A4/PXR metabolism in the hepatic cell line Huh7. This maturation induces primary hepatocyte characteristics to study CYP3A4 induction and the induction of other CYP450 enzymes, which are regulated by the nuclear receptor PXR.

Previously it has been shown that CDK2 pathway inhibition in 4-week confluently cultured Huh7s resulted in an increase in basal expression levels of CYP3A4 and PXR. Here, we devised a new method to mature the PXR dependent CYP3A4 machinery and tested different compounds to inhibit the CDK pathway. Aloisine A (an inhibitor of CDK1, CDK2 and CDK5 pathways), BML-259 (an inhibitor of CDK2 and CDK5 pathways), CDK1/2 inhibitor III (an inhibitor of CDK1 and CDK2 pathways) and dinaciclib (an inhibitor of CDK1, CDK2, CDK5, and CDK9 pathways) were incubated with Huh7s for 24 hrs. We only observed an increase in basal expression levels of CYP3A4 and PXR in the dinaciclib treated group (Fig. S1).

According to the literature, the observed phenotypic alteration of Huh7s can partially be explained by CDK2 inhibition^28,29^. However, only 2-fold change in basal CYP3A4 expression levels was achieved upon CDK2 knockdown via siRNA. In addition, in the same study, Huh7s were incubated with another CDK2 inhibitor, seliciclib, and basal CYP3A4 levels were increased by 3-fold, however, no induction was observed upon rifampicin treatment^29^. These findings suggest that the CDK2 pathway may not be the single player in the observed Huh7 maturation. Given the fact that dinaciclib is a very potent inhibitor of a wide array of different CDK pathways, the rapid maturation of this hepatic cell line could be the result of the inhibition of several cellular mechanisms involved in cell cycle^32^. Pictures of cells taken after 24 hr of treatment demonstrated that Huh7s stopped proliferating following dinaciclib treatment (Fig. S3). In our dose response study, we identified 20 nM as the optimal dinaciclib concentration, where the basal CYP3A4 and PXR expression levels were the highest (Fig. 2A). These results showed that we were able to achieve significantly higher expression levels via 24 hr dinaciclib treatment compared to 4-week confluent cultures (Fig. 2B).

Multiple studies have shown that increasing the availability of PXR increases the expression of downstream genes without the addition of exogenous ligands. Sivertsson *et al*. showed that confluent culturing of Huh7s increases the PXR levels and consequently, the basal levels of downstream genes without the addition of any drug^27,29^. In addition, it was shown that PXR can be activated by endogenous ligands such as steroids and many other endogenous compounds produced by the cell^33–35^. Thus, we hypothesize that in non-treated Huh7 cells there are more endogenous ligands than available PXR, making PXR a limiting factor. When dinaciclib increases the amount of available PXR, more PXR can be activated by endogenous ligands, resulting in an increase in basal CYP3A4 expression levels.

As the next step, we assessed the inducibility of CYP3A4 via rifampicin, which is a known inducer of this enzyme through PXR activation. A 5.4-fold induction was achieved for CYP3A4 at 20 μM rifampicin (Fig. 3), which is comparable to some of the primary human hepatocyte inducibility data following cryo-preservation^36,37^. On the other hand, non-treated Huh7s were not inducible as expected, as shown in Fig. 3A. In addition, we compared the inducibility of the dinaciclib pre-treated group to the 4-week confluent culture group (Fig. S2) and showed that the dinaciclib pre-treated group retained its inducibility and achieved higher fold induction levels. This data showed that dinaciclib treatment allowed for not only higher basal expression levels of PXR and subsequently CYP3A4, but it also allowed for a higher enzyme fold induction upon drug treatment. Interestingly, we observed an increase in PXR expression upon rifampicin treatment. While the induction of PXR expression through rifampicin treatment is not a known fact, we observed this outcome both in cell lines (Huh7, HepG2) and in hepatocyte-like differentiated iPSC cells^38^. The results from this study demonstrated that PXR expression levels were increased upon rifampicin treatment in hepatic cell lines and differentiated iPSCs. It is possible that ligand-bound PXR might be regulating its own expression by a transcriptional auto-regulatory feedback mechanism.

The transcription factor PXR regulates many CYP450 enzymes other than CYP3A4, including 2C9 and 2C19^2,39^. In addition, rifampicin has been shown to be a weak inducer of CYP1A2 in multiple studies, implying that PXR could be involved in the regulation of CYP1A2^40–42^. As such, we tested the usability of this robust platform to study other CYP450 enzymes. We compared the fold induction levels of these three enzymes in the non-treated control group and in the dinaciclib pre-treated Huh7 cultures (Fig. 4). The expression levels of all three enzymes were significantly induced upon rifampicin induction as demonstrated in Fig. 4B, whereas no inducibility was observed in the non-treated group. These data suggest that the use of matured Huh7s is not limited to CYP3A4 studies and can be applied to drug induction studies involving other CYP450 enzymes under the regulation of PXR. We also studied the inducibility of CYP2E1, which is not regulated by PXR, to confirm that the Huh7 maturation was specific to PXR-regulated CYP enzymes. As expected, no difference was observed in CYP2E1 induction among the non-treated and dinaciclib pre-treated groups.

Once we showed that dinaciclib treatment allows for the inducibility of PXR/CYP3A4 mechanism, we wanted to demonstrate the use of the matured Huh7s in DDI studies. Amiodarone is a drug used in the treatment of cardiac arrhythmias, which is metabolized to mono-N-desethylamiodarone and di-N-desethylamiodarone by CYP3A4, and can become toxic upon CYP3A4 induction^12,43–45^. Previously, the interaction between amiodarone and CYP3A4-inducer rifampicin was studied in HepG2 cells overexpressing CYP3A4^31^. It was shown that the DDI between rifampicin and amiodarone resulted in apoptosis in these cells as amiodarone became hepatotoxic in the presence of high CYP3A4 activity. In order to demonstrate this established DDI in our platform, we incubated the non-treated and pre-dinaciclib treated Huh7s with these two drugs, where we used 20 μM rifampicin and an amiodarone concentration range of 0 μM−50 μM (Fig. 5). Following incubation with amiodarone only, the dinaciclib-treated group demonstrated a decreased cell viability compared to the non-treated group, due to increased CYP3A4 metabolism following dinaciclib treatment. In the absence of dinaciclib treatment, we observed a decrease in cell viability with increasing amiodarone concentrations, however, the viability did not significantly vary between the amiodarone only and amiodarone/rifampicin groups (Fig. 5A). This indicates that without the dinaciclib treatment the CYP3A4 metabolism in Huh7s is not inducible and does not result in DDI. On the other hand, the cell viability was significantly decreased in matured Huh7s treated with both drugs at amiodarone concentrations of 12.5 μM and 25 μM, as a result of increased CYP3A4 metabolism via dinaciclib treatment (Fig. 5B). This effect was not observed in cells treated with 50 μM amiodarone. The combination of dinaciclib pre-treatment and 50 μM amiodarone administration could be already highly toxic to result in a significant difference in cell viability upon additional treatment with rifampicin.

In summary, we were able to induce primary hepatocyte characteristics in the hepatic cell line Huh7 via a potent CDK inhibitor, dinaciclib, in 24 hrs. Previously, such maturation of the CYP3A4/PXR metabolism required a 4-week confluent culture period which is impractical for many researchers as well as the pharmaceutical industry. Thus, the dinaciclib pre-treatment of Huh7s is a major advance for all researchers who do not have access to primary human hepatocytes yet want to study drug metabolism through CYP3A4 and other CYP450 enzymes regulated by PXR. In this study, we demonstrated both CYP450 inducibility and an example of CYP3A4-mediated DDI studies (rifampicin-amiodarone) in matured Huh7s. While further studies are required to elucidate the exact maturation mechanism, the presented results indicate the use of dinaciclib pre-treated Huh7s as a cost-effective platform, that can be rapidly developed and used for DDI and toxicology studies.

## Materials and Methods

### Huh7 culturing and seeding

The human hepatoma cell line (Huh7) was obtained from Creative Biolabs (Creative Biolabs, Shirley, NY, USA). The cells were cultured in Dulbecco’s modified eagle’s medium (DMEM, Life Technologies, Carlsbad, CA, USA) supplemented with 10% fetal bovine serum (FBS, Peak Serum, CO, USA), 200 U/mL penicillin, and 200 μg/mL streptomycin. The cells were seeded in 12 well plates (Corning, Corning, NY, USA) at a density of 2 × 10^5^ cells per well and incubated at 37 °C and 5% CO_2_. After the cells reached 100% confluency, they were maintained for an additional four weeks with frequent media changes. The confluency of the freshly-plated cells used in CDK inhibition studies was around 90%.

### CDK inhibitor treatment

Huh7 cells at 90% confluency were treated with aloisine A (0.5 μM and 1 μM), BML 259 (0.2 μM and 0.5 μM), CDK1/2 (1 nM and 10 nM) or dinaciclib (10 nM and 20 nM) in William’s medium E supplemented with 200 U/mL penicillin, 200 μg/mL streptomycin for 24 hrs. The media did not include FBS to avoid the interference of serum proteins. The experiments were performed in triplicate.

### Rifampicin treatment

After dinaciclib treatment or culturing after four weeks, the cells were treated with 0, 5, 10, or 20 μM rifampicin in supplemented William’s medium E for 24 hrs. All groups were exposed to 0.2% DMSO (vehicle). The media did not include FBS to avoid the interference of serum proteins.

### Amiodarone treatment and cell viability analysis

Huh7 cells were plated in 12 well plates at a density of 2 × 10^5^ cells per well and cultured in supplemented DMEM media. All of the subsequent drug treatments were performed in supplemented William’s medium E. 24 hrs after plating, the cells were treated with 20 nM dinaciclib or the corresponding volume of DMSO (0.2%) for 24 hrs. Then, the cells were treated with 20 μM rifampicin or the corresponding volume of DMSO (0.2%) for 8 hours to induce an initial response to rifampicin before exposure to amiodarone. The cells were then treated with 0, 12.5, 25 or 50 μM of amiodarone hydrochloride (Sigma-Aldrich) with an additional 20 μM rifampicin or the corresponding volume of DMSO (0.2%) during 24 hrs. Finally, cell viability was assessed using PrestoBlue^TM^ Cell Viability Reagent (Thermo Fisher Scientific, Waltham, MA, USA) according to the manufacturer’s instructions.

### RNA isolation and RT-PCR analysis

At the end of the rifampicin treatment, the cells were lysed using trizol reagent (Thermofisher Scientific). The RNA of each sample was isolated and purified using PureLink RNA Mini kit (Thermofisher Scientific) according to the manufacturer’s instructions. A total of 800 ng of RNA were reverse transcribed using a cDNA synthesis kit (iScript, Bio-rad, Portland, ME, USA) according to the manufacturer’s instructions. The cDNA obtained was used for quantitative PCR using Power SYBR Green PCR master mix kit (Life Technologies, Carlsbad, CA, USA). The reaction was performed in a ViiA 7 Real-time PCR system (Life Technologies) following the manufacturer’s instructions. The primers for PXR, CYP3A4, CYP1A2, CYP2C9, CYP2C19, CYP2E1 and the housekeeping genes PSMB2 and EIF1 are given in Table S1. All primers were validated and exhibited a single clear peak after melting curve analysis (Fig. S4). The relative mRNA expression was quantified using the comparative Ct (ΔΔCt) method.

### Statistical analysis

All quantitative data are presented as the mean ± standard error of the mean (SEM). GraphPad Prism software (Graphpad Software Inc., San Diego, CA) was used for statistical analysis of qPCR results. Statistical significance of the results was assessed using one-way ANOVA. P-values equal or less than 0.05 were considered statistically significant.

## Supplementary information

SUPPLEMENTARY INFORMATION
