# Supplementary material for: Rapid maturation of the hepatic cell line Huh7 via CDK inhibition for PXR dependent CYP450 metabolism and induction
Source: Sci Rep. 2019 Nov 1;9:15848. doi: 10.1038/s41598-019-52174-w (PMC6825149; doi:10.1038/s41598-019-52174-w)
Supplement: Supplementary file 1 — SUPPLEMENTARY INFORMATION [file 41598_2019_52174_MOESM1_ESM.pdf]

SUPPLEMENTARY INFORMATION

Rapid maturation of the hepatic cell line Huh7 via CDK inhibition for PXR dependent CYP450 metabolism and induction

Beyza Bulutoglu[1,#], Safak Mert[1,#], Camilo Rey-Bedón[1], Sarah L. Deng[1], Martin L. Yarmush [1,2,*], O. Berk Usta[1,*]

[1] Center for Engineering in Medicine, Massachusetts General Hospital, Harvard Medical School and Shriners Hospitals for Children, Boston, MA, 02114, USA

[2] Department of Biomedical Engineering, Rutgers University, Piscataway, NJ, 08854, USA

[#] These authors contributed equally.

[*] Correspondence and material requests should be addressed to M.L.Y. and O.B.U. (email: berkusta@gmail.com; ousta@mgh.harvard.edu; ireis@sbi.org).

Supplementary Figures

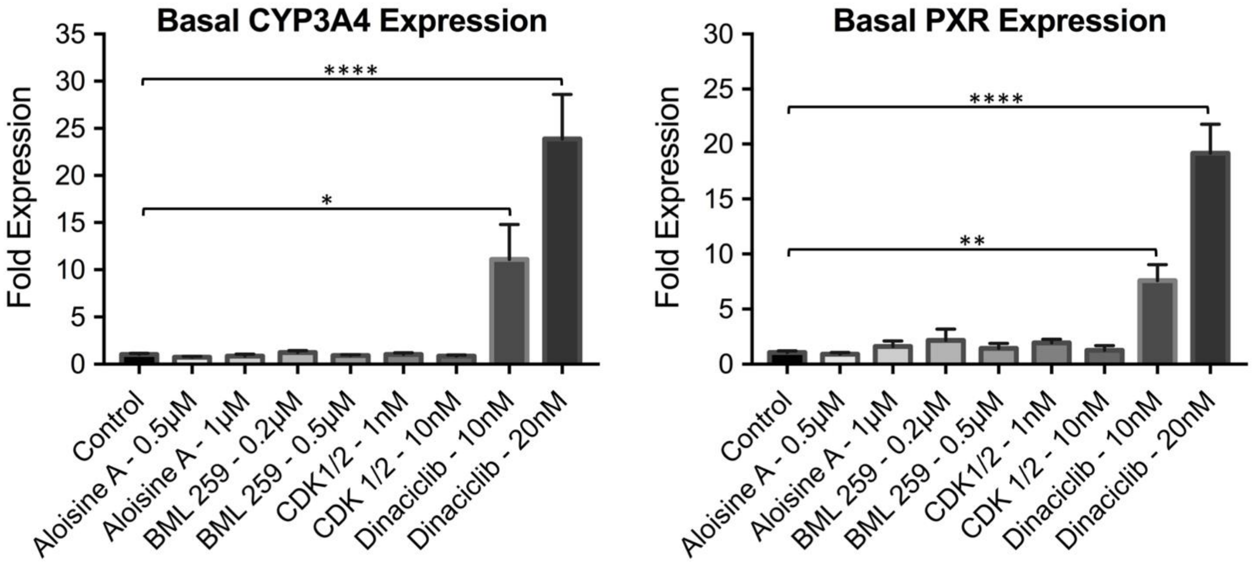

Figure S1. Change in basal CYP3A4 and PXR expression levels in Huh7s incubated with different CDK inhibitors. Huh7 cells were incubated with different concentrations of aloisine A, BML 259, CDK ½ inhibitor III and dinaciclib based on their reported IC50 values. Following 24 hrs incubation, the changes in the mRNA levels of CYP3A4 and PXR were quantified via quantitative real-time PCR. The data are presented as mean ± SEM. N=3, * = $p \leq 0.05$, ** = $p \leq 0.01$, **** = $p \leq 0.0001$.

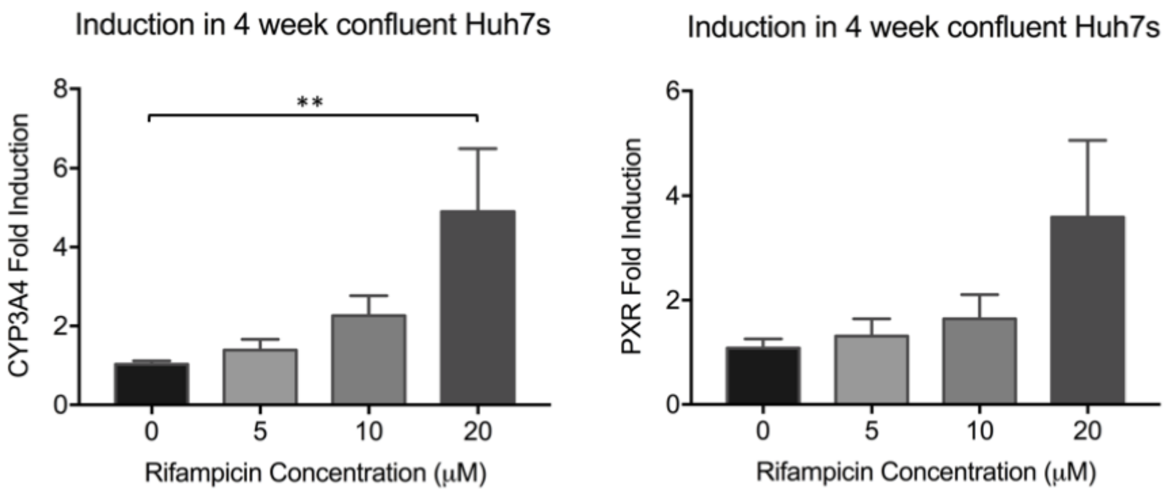

Figure S2. The fold induction levels of CYP3A4 and PXR following rifampicin treatment in 4-week confluent Huh7 cultures. Confluently cultured Huh7 cells were induced with varying concentrations of rifampicin at the end of 4 weeks. CYP3A4 and PXR levels were quantified via quantitative real-time PCR. The data are presented as mean ± SEM, N=6, ** = $p \leq 0.01$.

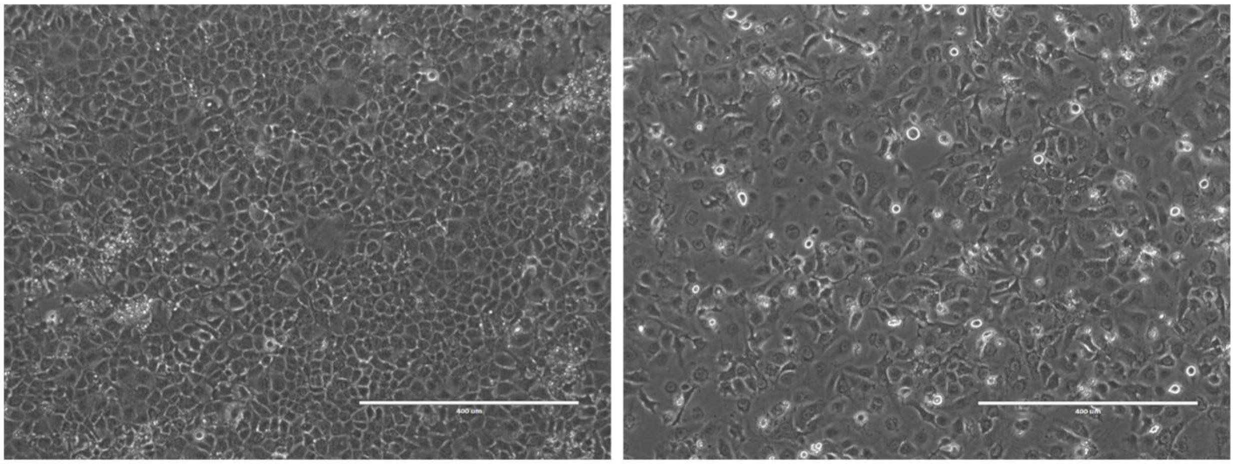

Figure S3. Bright field images of Huh7s. Same number of cells were seeded in 12 well plates. One group did not receive dinaciclib treatment (left) and the other one was treated with 20 nM dinaciclib for 24 hrs (right). The pictures were taken at the end of 24 hrs following the treatment, which show that Huh7s stopped proliferating in the dinaciclib-treated group.

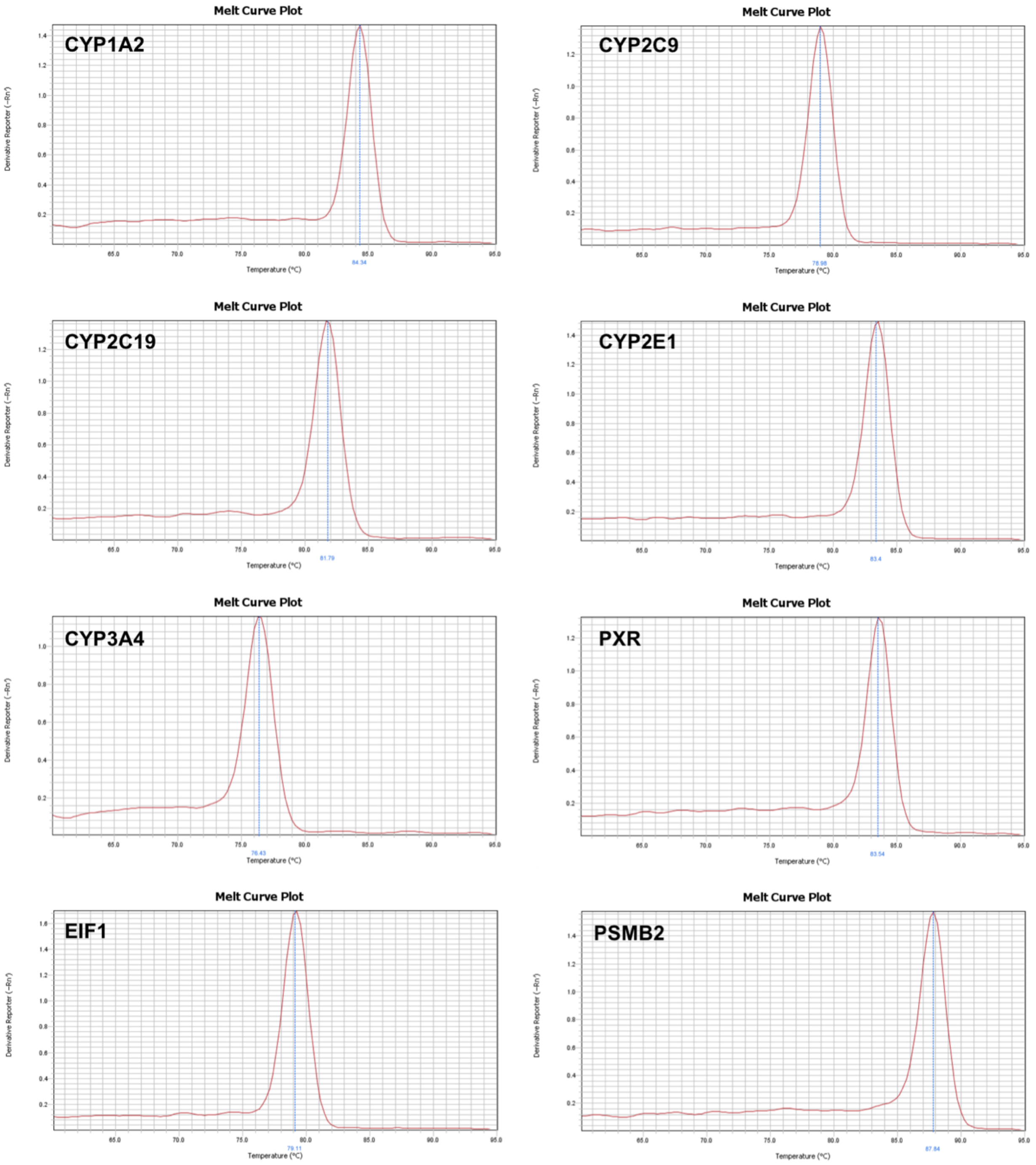

Figure S4. Melting curve analyses of qPCR primers. All primers were validated and exhibited a single clear peak.

Supplementary Tables

Table S1. Sequences of qPCR Primers

Target	Forward primer	Reverse primer
**hPXR**	5’ AGCTTTCCCACCCTCTTTGG 3’	5’ CTGAACAGTGTGCTCTGGGG 3’
**CYP3A4**	5’AATCACTGTTGGCGTGGGG 3’	5’ AATGGGCAAAGTCACAGTGGA 3’
**CYP1A2**	5’ CTTCGGACAGCACTTCCCTG 3’	5’ AGGGTTAGGCAGGTAGCGAA 3’
**CYP2C9**	5’ GCAGCTCTCTTTCCTCTGGG 3’	5’ GCACAGTGAAACATAGGAAACTCTC 3’
**CYP2C19**	5’ TGAAGAAGCACAGATGGTCTGG 3’	5’ GACGGGTCAGAAGAAGCATCA 3’
**CYP2E1**	5’ GACTGCCTGCTCGTGGAAAT 3’	5’ CTCTGTCCCCGCAAAGAACA 3’
**PSMB2**	5’ GCTCTGAGGTGCTGTCTCAC 3’	5’ GTCGGAGGCGACAAGAACAT 3’
**EIF1**	5’ AACCATTTGGGGTCCGCTTT 3’	5’ GCGCCTATTGCTTGACCTCT 3’
